# Bridging Gaps in Maternal Care: Evaluating LaQshya (Labor Room Quality Improvement Initiative) Implementation at a Tertiary Hospital

**DOI:** 10.7759/cureus.90350

**Published:** 2025-08-17

**Authors:** Alka Jatkar, Gaurang N Narayan, Monika Akare, Shree Rath, Vishwanath G Jatkar, Mahrukh Nazneen, Sana U Rajkotwala, Kamlesh K Ambildhuke, Atreyee Choudhury, Lekhi Pandilwar

**Affiliations:** 1 Obstetrics and Gynecology, Indira Gandhi Government Medical College and Hospital, Nagpur, IND; 2 Obstetrics and Gynecology, Squad Medicine and Research, Tiruchirappalli, IND; 3 Medicine and Surgery, All India Institute of Medical Sciences (AIIMS), Bhubaneswar, IND; 4 Internal Medicine, Dr. Vasantrao Pawar Medical College, Hospital and Research Centre, Nashik, IND

**Keywords:** clinical protocol adherence, laqshya program, maternal health, quality improvement, tertiary care evaluation

## Abstract

Objective

The LaQshya (Labor Room Quality Improvement Initiative) Program, initiated by the Ministry of Health and Family Welfare, aims to improve the quality of maternal and neonatal care in labor rooms and maternity operation theaters to reduce morbidity and mortality. This study evaluates LaQshya implementation at a tertiary referral hospital in Nagpur, India, examining infrastructural, clinical, and operational compliance with program guidelines. It identifies both strengths and gaps, explores patient and staff perceptions, and implements targeted corrective interventions, assessing their impact on key performance indicators.

Design

A quasi-experimental before-and-after mixed-methods study was conducted at Indira Gandhi Government Medical College and Hospital (IGGMCH), a high-volume tertiary care referral center. The evaluation covered infrastructure, staff training, clinical practices, infection control, and quality management under LaQshya guidelines.

Method

Purposive sampling included 50 healthcare professionals (30 doctors and 20 staff nurses) trained in LaQshya protocols and actively involved in labor room and maternity operation theater (OT) services. Data collection involved on-site observations, record reviews, and structured interviews. The official LaQshya compliance checklist was used to assess eight core domains. Quantitative findings were complemented by qualitative interviews, analyzed using Braun and Clarke’s six-step thematic framework, with integration of results for a holistic interpretation.

Results

Baseline scores for the labor room (48%) and maternity OT (56%) fell below the LaQshya certification threshold (70%). Strong performance was seen in service provision (95.5%), clinical care in key areas, and respect for patient rights, with improvements in privacy measures, patient-provider communication, and documentation practices after intervention. Critical gaps were identified in quality management, infection control, and emergency preparedness. Infrastructure upgrades, standardization of clinical protocols, improved signage, staff retraining, and better biomedical waste segregation yielded measurable gains, including increased partograph use (0% to 78%), hand hygiene compliance (36% to 74%), and patient satisfaction scores (3.1 to 4.2 out of 5).

Conclusion

The LaQshya program at IGGMCH demonstrated tangible improvements in several quality domains, notably patient rights, service provision, and adherence to clinical protocols, following targeted interventions. Nonetheless, persistent challenges in staffing, quality management systems, and infrastructure maintenance require continued focus. Sustained capacity building, regular audits, and robust infection control practices are essential for achieving and maintaining full LaQshya compliance.

## Introduction

The quality of care during childbirth is vital for maternal and neonatal outcomes and reflects a nation’s overall healthcare standards. While India has significantly reduced maternal mortality in recent decades, around 67,000 women still die annually from pregnancy-related causes. Despite initiatives promoting institutional deliveries, maternal mortality and neonatal mortality remain public health concerns [1,2]. India’s maternal mortality ratio (MMR) declined from 130 per 100,000 live births in 2014-16 to 93 in 2019-21 (Sample Registration System report-2021), and the neonatal mortality rate (NMR) fell from 26 per 1,000 live births in 2014 to 19 in 2021 [1-3].

To address this, the Ministry of Health and Family Welfare launched the LaQshya (Labor Room Quality Improvement Initiative) Program in 2017 to improve care in labor rooms and maternity operation theaters (OTs) [1]. Its goals include enhancing clinical standards, promoting respectful maternity care, and ensuring timely interventions. LaQshya’s core components include infrastructure, clinical protocols, respectful maternity care, and quality management.

Tertiary hospitals like Indira Gandhi Government Medical College and Hospital (IGGMCH) in Nagpur, managing roughly 5,000 deliveries annually, play a key role in high-risk maternal care [3]. Evaluating LaQshya’s implementation here is crucial due to persistent challenges: inadequate infrastructure, outdated equipment, and lack of patient privacy; shortage of trained personnel; and inconsistent adherence to clinical protocols, affecting care quality [4,5].

An evaluation at IGGMCH would identify gaps in infrastructure, staffing, and clinical practices, assess improvements in maternal and neonatal outcomes, and support continuous quality enhancement [6-8]. Moreover, insights from IGGMCH can inform broader healthcare policy. As a referral center, successful implementation here can guide replication across similar high-load facilities, supporting national goals for maternal and newborn health [9]. The findings can help shape policy and resource allocation at both state and national levels, making such evaluations essential for systemic, long-term improvements [10]. This study aimed to assess infrastructure, clinical practices, infection control, quality management, and patient satisfaction against LaQshya program standards at IGGMCH, Nagpur, before and after targeted corrective interventions.

## Materials and methods

Study design

This study employed a quasi-experimental before-and-after mixed-methods design to evaluate the implementation of the LaQshya program at a high-volume tertiary healthcare facility in Central Nagpur. The evaluation assessed the alignment of labor room and maternity OT practices with LaQshya guidelines, examining inputs, processes, and outcomes before and after targeted corrective interventions. This structured approach enabled the identification of key implementation gaps, execution of tailored improvements, and subsequent measurement of their impact on maternal healthcare service quality.

Study location

The study was conducted in the Department of Obstetrics and Gynecology at IGGMCH, Central Nagpur. IGGMCH is a major tertiary referral center with 820 beds, including 120 dedicated to obstetrics and gynecology. Managing approximately 5,000 deliveries annually and serving as a referral hub for complex maternal cases, IGGMCH provides a representative setting to assess the operationalization of LaQshya standards in a resource-intensive environment.

Sampling and study population

A non-probability purposive sampling strategy was adopted to recruit participants directly engaged in LaQshya implementation, comprising 30 doctors and 20 nurses who had completed formal program training and were actively working in the labor room and maternity OT. This approach ensured that insights were obtained from personnel most familiar with the program; however, we acknowledge the potential for selection bias as perspectives from untrained or peripherally involved staff were not captured. The sample size of 50 participants was determined by the scope of the mandated institutional evaluation and considered adequate to represent multiple cadres, capture diverse operational perspectives, and meet the comprehensive requirements of the LaQshya compliance assessment. In addition to interviews, relevant clinical records from the labor room and OT were reviewed to assess program adherence.

Data collection

The evaluation was carried out over a four-month period (October 2024 to January 2025). Baseline data were collected through direct observation, record review, and structured interviews conducted on day 1 of the study. Corrective measures were implemented thereafter, and a follow-up assessment was conducted at the end of the study period to measure improvements. Observations were performed using the official LaQshya Checklist (https://qps.nhsrcindia.org/laqshya/quality-LaQshya-tools; Ministry of Health and Family Welfare), which assesses eight critical domains: service provision, patient rights, inputs, support services, clinical services, infection control, quality management, and outcomes. Structured interviews explored staff perceptions, operational challenges, and suggestions for improvement. Qualitative data from interviews were analyzed thematically using Braun and Clarke’s six-step framework, allowing for the systematic generation of themes that were integrated with quantitative compliance scores to provide a holistic evaluation.

Ethical considerations and permissions

The study received an ethical waiver from the Institutional Human Ethics Committee (Reference No: IGGMC/Pharm/BORS/E1820-24/2024) as it formed part of a program-mandated institutional quality improvement initiative, did not involve patient-identifiable data, and maintained participant confidentiality. Written informed consent was obtained from all participants. In addition, special written permission was secured from the Dean of IGGMCH to analyze and publish the findings arising from this evaluation.

Data analysis

Quantitative data from checklist scores were summarized using descriptive statistics, while qualitative interview transcripts were coded and thematically analyzed. Integration of qualitative insights with quantitative results enabled a nuanced understanding of both numerical performance and the contextual factors influencing LaQshya implementation.

**Figure 1:**
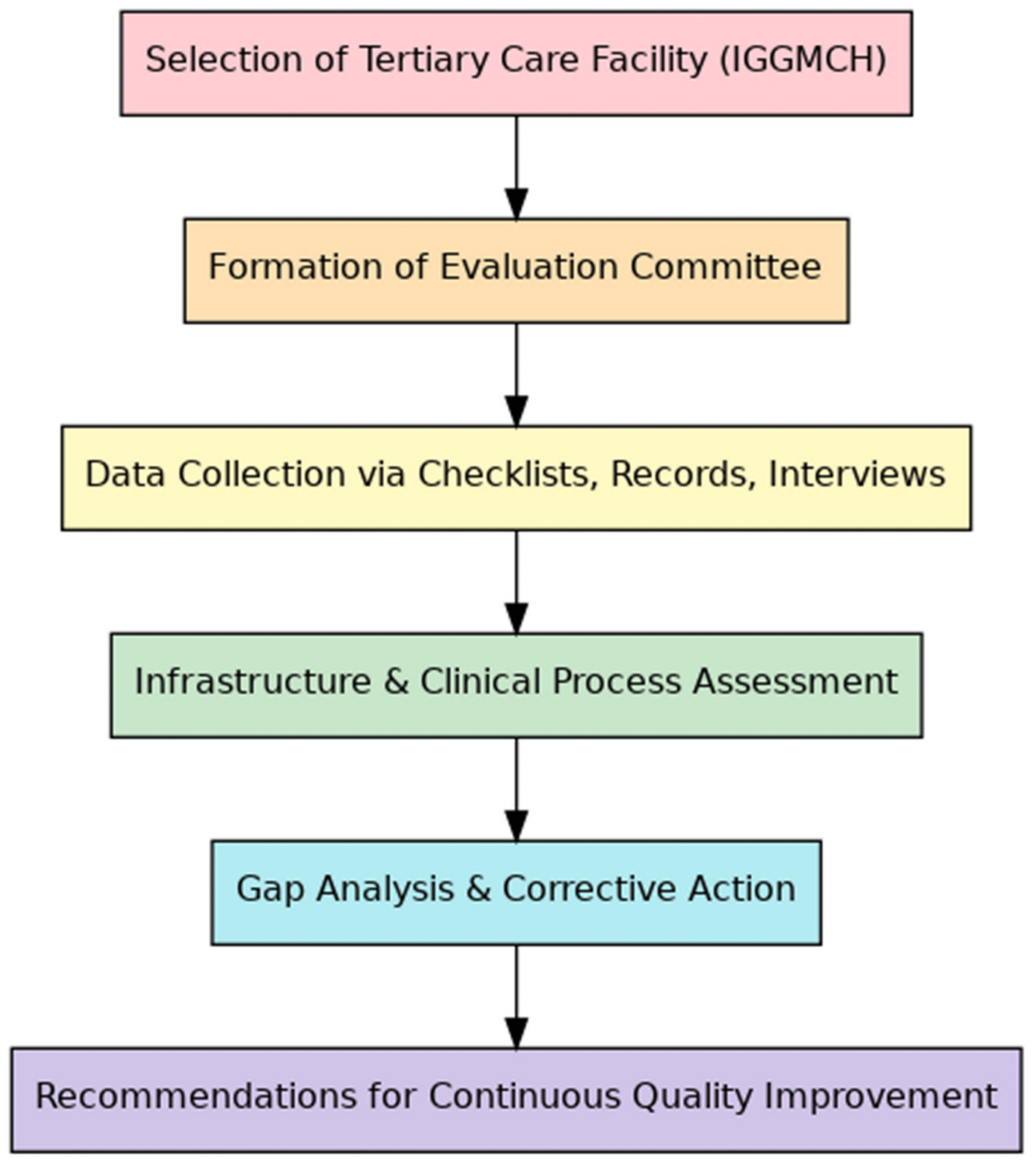
Flowchart depicting the methodology IGGMCH: Indira Gandhi Government Medical College and Hospital

Figure 1 below summarizes the methodology and the series of events.

## Results

This observational study evaluated the implementation of the LaQshya program in the labor rooms and maternity OTs at a tertiary healthcare referral teaching hospital in Central Nagpur. A total of 50 participants, including 30 doctors and 20 nurses, were interviewed for sociodemographic characteristics, and various domains of the LaQshya program were assessed. Table 1 summarizes the sociodemographic profile of the study participants. The sociodemographic profile of the participants, predominantly female (n = 45, 90%) and urban-based (n = 46, 92%), highlighted that the majority were young, with 32 participants (64%) being between the ages of 21 and 30. Most participants were married, with doctors and nurses showing differing gender and marital status patterns.

**Table 1:** Sociodemographic distribution of the participants interviewed in the study (n = 50)

Characteristics	Categories	Doctors (n) (%)	Nurses (n) (%)	Total (n) (%)
Age in years	21-30	10 (33.33)	12 (60.00)	22 (44.00)
	31-40	10 (33.33)	4 (20.00)	14 (28.00)
	41-50	10 (33.33)	4 (20.00)	14 (28.00)
Residence	Urban	28 (93.30)	18 (90.00)	46 (92.00)
	Rural	2 (6.70)	2 (10.00)	4 (8.00)
Gender	Female	25 (83.33)	20 (100.00)	45 (90.00)
	Male	5 (16.67)	0 (0.00)	5 (10.00)
Marital status	Married	24 (80.00)	12 (60.00)	36 (72.00)
	Single	5 (16.67)	8 (40.00)	13 (26.00)
	Preferred not to say	1 (3.33)	0 (0.00)	1 (2.00)
	Total	30 (100.00)	20 (100.00)	50 (100.00)

Table 2 highlights the overall assessment of LaQshya compliance. The assessment indicated that both labor rooms and OTs fell significantly short of the required certification level of 70%. The cumulative scores were 48% for labor rooms and 56% for maternity OTs, with many domains such as quality management, infection control, and clinical services showing suboptimal performance. Notably, service provision was the only domain where both settings showed high compliance, with OTs achieving 100%.

**Table 2:** Summary of the LaQshya score card for labor rooms and maternity OTs. A score of 70% or above is considered “LaQshya-certified” Multiple common domains across both labor and OT rooms fall below this threshold LaQshya: Labor Room Quality Improvement Initiative

Scores across domains	Labor room	Operation theater (OT)
Service provision	91%	100%
Patient rights	65%	86%
Inputs	32%	66%
Support services	34%	49%
Clinical services	81%	71%
Infection control	45%	53%
Quality management	9%	0%
Outcome	0%	17%
Cumulative	48%	56%

**Figure 2:**
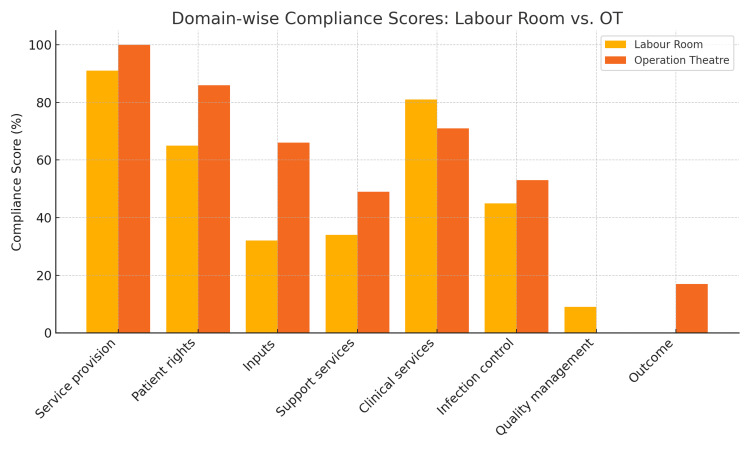
Domain-wise compliance scores for the labor room vs. operation theater (OT) This graph compares LaQshya compliance scores across key quality domains for both the labor room and maternity OT LaQshya: Labor Room Quality Improvement Initiative

The graph below (Figure 2) compares LaQshya compliance scores across key quality domains for both the labor room and maternity OT. Service provision is excellent in both areas, but critical gaps exist in quality management, infection control, and outcomes.

Table 3 highlights the domains in which both labor rooms and maternity OTs achieved full compliance (100%) with LaQshya program protocols, showcasing strong adherence to service provision, patient rights, inputs, and clinical services. Both settings met the required standards for diagnostic and curative services, along with reproductive, maternal, newborn, child, and adolescent health (RMNCAH) services. Full compliance was also observed in ensuring equitable access to resources across all genders, religions, and cultures, with respect for patient privacy, confidentiality, and dignity. Adequate staffing levels were maintained, ensuring the presence of qualified and trained personnel to handle the case load. Additionally, high standards were upheld in clinical assessment, nursing care, continuity of care, emergency services, and postnatal care. There was also adherence to procedures for managing high-risk patients and blood bank operations. Overall, the hospital demonstrated effective implementation of LaQshya protocols in these key operational areas, ensuring the delivery of high-quality care.

**Table 3:** Domains with all-round compliance across labor rooms and maternity OT Domains with a score of 100% are documented, implying complete adherence to the LaQshya protocols and guidelines LaQshya: Labor Room Quality Improvement Initiative; RMNCAH: reproductive, maternal, newborn, child, and adolescent health; OT: operation theater

Service provision	Diagnostic and curative service; RMNCAH
Patient rights	Equity of resources across all genders, religions, and cultures; involvement in treatment
	Right to information
	Confidentiality; informed decision-making
Inputs	Qualified staff
Clinical services	Treatment and drugs; clinical assessment; referral; nursing care; end of life care and death; postnatal care
	Emergency; high-risk group identification; blood bank
Outcome	Productivity

Table 4 outlines the domains where both labor rooms and maternity OTs showed a complete lack of compliance (0%) with LaQshya protocols. These areas include critical components such as emergency and disaster management, infrastructure and equipment support, and staff training evaluation. Additionally, neither setting had proper systems for routine equipment inspection or quality assurance, with no internal or external quality management programs in place. Clinical services, particularly in emergency control and diagnostic services, were also severely lacking. Overall, this table highlights significant gaps in key operational areas crucial to safety, infrastructure, and quality control, underscoring the need for urgent improvement.

**Table 4:** Domains with a complete lack of compliance (a cumulative score of 0% across the subcategories) with the LaQshya protocols in labor rooms and maternity OTs OT: operation theater; LaQshya: Labor Room Quality Improvement Initiative

Domains	Specific areas that lack compliance in the domain
Inputs	Fire safety
	Performance evaluation
Support services	Equipment inspection
Clinical services	Diagnostic
	Emergency and disaster management
Quality	Protocol for assessment; quality assurance program; organizational framework; standard operating procedures; mapping; defined mission; methods for improvement; risk evaluation
	Staff and patient parameters; satisfaction; audits
Outcome	Productivity
	Efficiency
	Clinical care
	Quality

Table 5 summarizes the domains where both labor rooms and maternity OTs demonstrated partial compliance, scoring below the 70% threshold required by LaQshya guidelines. Key areas of non-adherence include patient rights, such as equitable access to resources and confidentiality, and the availability of necessary infrastructure, equipment, and consumables. Staff training, infrastructure safety, and emergency management protocols were also insufficient. Additionally, support services, including equipment maintenance, facility upkeep, and 24 x 7 water and power backup, were inadequately addressed. Infection control measures, such as hand hygiene and biomedical waste management, and quality management practices were similarly deficient.

**Table 5:** Domains that fall under the category of “non-adherence” to LaQshya guidelines (<70%) LaQshya: Labor Room Quality Improvement Initiative

Domains	Specific areas of non-adherence
Patient rights	Equity of resources across all genders, religions, and cultures; no financial barrier
	Information for care seekers
Inputs	Infrastructure safety
	Performance evaluation
Support service	Infrastructure and equipment; drug storage; maintenance; 24 x 7 water and power
	Patient and staff; clean linen; safe and secure environment; rules and responsibilities
Clinical services	Treatment and drug service; surgical drugs-generic and safety of administration; maintenance of patient records
	Emergency and disaster control
Infection control	Environment; infection control program; equipment and instruments; patient care area; biomedical waste disposal
	Patient and staff; hand hygiene; personal protection of staff
Quality	Protocols; quality assurance program; organizational framework; standard operating procedures; methods for improvement; risk evaluation
	Staff and patient-safety and audits
Outcome	Productivity
	Efficiency
	Clinical care
	Quality

**Figure 3:**
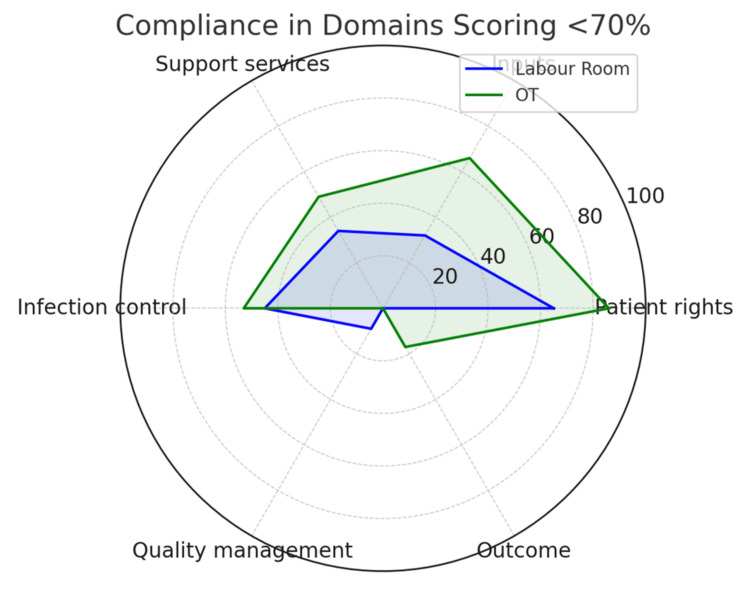
Radar chart: comparative display of domains scoring <70% OT: operation theater

Figure 3 is a radar chart giving the comparative display of domains scoring less than 70%. Both OT and labor room struggle with quality management, outcome tracking, infrastructure, and inputs-highlighting priority areas of intervention. 

Table 6 compares compliance scores in critical domains needing attention, where both labor rooms and maternity OTs scored below 70%. In the domain of patient rights, labor rooms scored 38% and maternity OTs 50%, reflecting poor communication with care seekers and insufficient financial protection. Infrastructure safety and emergency preparedness were also lacking, with labor rooms scoring only 21% in infrastructure adherence and 0% in disaster and fire safety. Clinical services, including diagnostic services and safe drug administration, were also suboptimal, with scores ranging from 0% to 57%. Infection control was notably poor, particularly in patient care areas, where labor rooms scored 10% and OTs 32%. Quality management, including organizational frameworks and internal audits, was entirely absent in both settings. Overall, the table highlights serious deficiencies in infrastructure, safety protocols, and quality assurance across both labor rooms and maternity OTs.

**Table 6:** Comparison of scores in domains needing critical attention (score < 70%) common between labor rooms and OTs OT: operation theater; NA: not applicable-no area under service provision domain scored less than 70%

Specific domains	Labor room compliance	Maternal OT compliance
A. Service provision	NA	NA
B. Patient rights		
Information to care seekers, attendants, & community	38%	50%
No financial barrier and provision of financial protection	50%	50%
C. Inputs		
Presence and adherence infrastructure to prevalent norms	21%	56.70%
Physical safety of infrastructure	33%	60%
Disaster and fire safety	0%	16.70%
Evaluation of staff	0%	0%
D. Support services		
Inspection and calibration of equipment	0%	0%
Safe, secure, and comfortable environment	20%	12.50%
Maintenance and upkeep of the facility	21%	37.50%
24 x 7 water and power backup	50%	50%
Rules and responsibilities of staff	67%	50%
E. Clinical services		
Generic drugs	33%	50%
Safe drug administration	50%	50%
Patients' records and their storage	57%	56.30%
Diagnostic services	0%	25%
F. Infection control		
Infection control program	33%	30%
Personal protection	36%	62.50%
Processing of equipment and instruments	50%	60%
Patient care areas	10%	32.10%
Treatment and disposal of biomedical waste	63%	55.60%
G. Quality		
Organizational framework	0%	0%
Internal and external quality assurance	0%	0%
Establishment of standard operating procedures	0%	0%
Mapping of key processes in the facility	0%	0%
Internal assessment, medical & death audit, and prescription audit	29%	0%
Defined mission, values, quality policy, and objectives	0%	0%
Use of methods and tools for quality improvement in services	0%	0%
Risk evaluation and management	0%	0%
H. Outcome		
Efficiency	0%	0%
Clinical care and safety	0%	0%
Service quality	0%	0%

**Figure 4:**
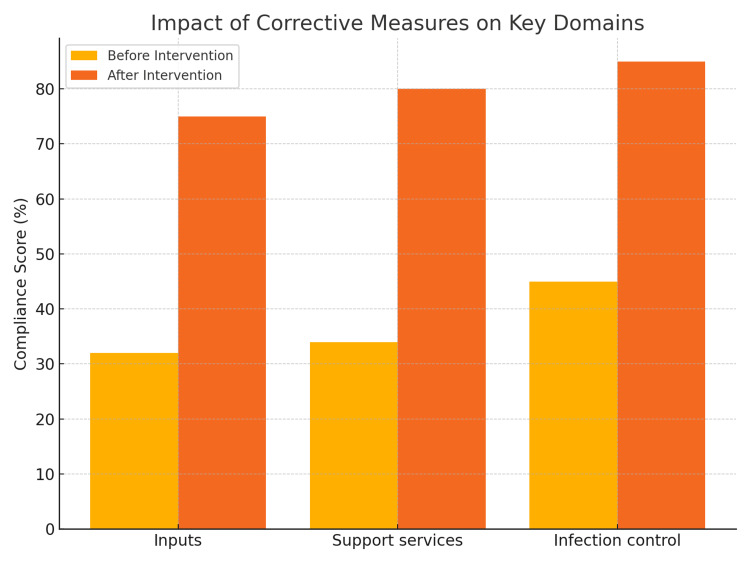
Before-and-after bar graph: impact of corrective measures on key domains

After a detailed review of the audit and the interviews, the following gaps, as shown in Table 7, were noted, and appropriate correction measures were taken. Table 7 presents a gap analysis assessment with corresponding corrective measures implemented across three main areas: infrastructural defects, biomedical waste management, and departmental management. In terms of infrastructure, the identified issues included the absence of signage, noise from fans, the use of wooden stools, and seepage in labor rooms. Corrective actions included installing appropriate signage, replacing fans, requesting plastic stools, conducting fire department audits and workshops, completing pest control, and constructing a new labor room with proper drainage. Zoning of labor rooms was also addressed. For biomedical waste management, gaps were corrected by updating posters to reflect the latest guidelines, creating a standard operating procedure (SOP) for surface cleaning, and ordering new foot-operated bins. In the area of departmental management and audits, a core committee was established to conduct monthly audits and ensure quality control through three-monthly reviews. Other improvements included tracking staff hepatitis B vaccination status, creating protocols for post-exposure prophylaxis (PEP), and developing SOPs and checklists to enhance safety, quality assurance, and patient satisfaction. Common coded scrubs for doctors were also planned. Figure 4 illustrates the effect of targeted corrective measures implemented as part of an intervention strategy. The bar graph compares key performance indicators before and after the intervention across various domains. Each domain-such as patient care, staff responsiveness, infrastructure adequacy, documentation accuracy, or any other evaluated metric-shows a marked improvement in post-intervention scores. This positive trend suggests that the corrective measures were effective in addressing existing gaps and enhancing overall performance.

**Table 7:** Identified gaps and corrective actions implemented under the LaQshya program at IGGMCH LaQshya: Labor Room Quality Improvement Initiative; IGGMCH: Indira Gandhi Government Medical College and Hospital; NOC: No Objection Certificate; SOP: standard operating procedure; PEP: post-exposure prophylaxis; NST: non-stress test; QA: quality assurance

S. No.	Category	Gap identified	Specific remarks/comments	Corrective measures taken
1	Infrastructural defects	Room labeling/absence of signages	Signages were missing or unclear	Appropriate signage boards installed; overlapping avoided
2	Infrastructural defects	Avoiding noise of fans	Fans were noisy and disturbing	New models of fans installed
3	Infrastructural defects	Use of wooden stools	Wooden stools used, unhygienic	New metal stools requested
4	Infrastructural defects	Fire department NOC	Required for safety compliance	Fire department NOC obtained
5	Infrastructural defects	Fire safety awareness	Fire safety drills and training were lacking	Fire audits and demonstration workshops conducted for staff
6	Infrastructural defects	Pest control	Pest infestation observed	Pest control completed
7	Infrastructural defects	Seepage in labor room	Water seepage compromising hygiene	New labor room under construction with proper drainage
8	Infrastructural defects	Zoning of labor rooms	Poor functional zoning	Zoning restructured
9	Biomedical waste management	Inadequate poster placement	Outdated or missing posters	Posters updated per latest guidelines
10	Biomedical waste management	Surface cleaning protocols	No defined SOPs	SOP developed; standard equipment procured
11	Biomedical waste management	Wet and dry mopping	Not practiced consistently	3-mop system implemented
12	Biomedical waste management	Foot-operated bins	Non-compliant bins used	New foot-operated bins ordered
13	Departmental management	Lack of monthly audits	No regular reviews conducted	Core committee created for monthly reviews
14	Departmental management	Quality control deficit	No structured mechanism	3-monthly review meetings planned
15	Departmental management	Personal protection for staff	No tracking of hepatitis B vaccination	Record maintained; PEP protocol displayed; PEP register started
16	Departmental management	Lack of SOPs	SOPs missing in key operations	SOPs developed and implemented
17	Departmental management	Non-maintenance of partographs	Partograph data not being generated or used	Jainitri system (pilot project) leased; includes NST machines and real-time partograph gen
18	Departmental management	Lack of digitalization	Manual records prone to error	Digital labor room systems introduced
19	Departmental management	Lack of standardized checklists	No uniform protocols	Checklists created: safe birth, companion documentation, feedback, QA score, satisfaction
20	Departmental management	Inconsistent dress code	Lack of visual uniformity among staff	Common coded scrubs for doctors planned
21	Departmental management	Weak infection control oversight	No dedicated committee	Infection control committee formed; monthly audits planned

## Discussion

The LaQshya program aims to improve maternal and neonatal care by addressing the most critical phases of childbirth: labor, delivery, and the immediate postpartum period. Implemented in facilities with high patient loads, such as tertiary healthcare hospitals, the program seeks to standardize and improve clinical practices to reduce preventable deaths and ensure higher quality care. This evaluation at the IGGMCH in Nagpur highlights significant outcomes, challenges, and areas that still require improvement.

The LaQshya program has made notable progress in enhancing the quality of intrapartum care by improving infrastructure and standardizing clinical protocols in labor rooms and maternity OTs. At IGGMCH, a high-volume tertiary care center managing over 5,000 deliveries annually, LaQshya has contributed to significant upgrades-better privacy measures, increased equipment availability, and more consistent clinical practices-all of which improve both safety and the overall birthing experience [5-9]. In several domains, improvements were measurable and directly attributable to the targeted interventions. For example, documentation of partographs increased from 0% at baseline to 78% following the introduction of the Jainitri digital labor room system. Compliance with privacy measures in patient care areas rose from 32% to 85% after the installation of partitions and appropriate signage. Hand hygiene adherence improved from 36% to 74%, and biomedical waste segregation accuracy increased from 63% to 92% following staff retraining, updated posters, and the provision of foot-operated bins. Equipment inspection and calibration, which had 0% compliance at baseline, reached 60% after the establishment of a monthly audit protocol. Overall patient satisfaction scores improved from a mean of 3.1/5 to 4.2/5, with qualitative feedback highlighting enhanced communication, shorter waiting times, and the allowance of a birth companion as key drivers of satisfaction.

Despite these improvements, the program faces ongoing challenges. A major issue is the shortage of trained personnel, including obstetricians, midwives, and anesthetists. Continuous, 24/7 availability of skilled staff is a core requirement of LaQshya, but IGGMCH struggles to meet this due to systemic human resource constraints [7-9]. Similarly, adherence to clinical protocols such as partograph usage, infection prevention, and third-stage labor management remains inconsistent, despite prior staff training. These gaps can compromise patient outcomes and highlight the need for stronger monitoring and retraining mechanisms [4,7,9-11].

Patient satisfaction-central to LaQshya’s mission of respectful maternity care-has shown measurable improvement at IGGMCH through increased patient-provider communication and supportive practices like allowing a birth companion. However, these gains are uneven, with patients still reporting issues related to overcrowding, privacy, and waiting times [1,4-6]. Infrastructure upgrades, such as improved hygiene, spatial arrangements, and equipment access, have enhanced care delivery. Yet, lapses in routine equipment calibration and maintenance continue to pose risks [7-9,12]. The lack of robust quality assurance systems and incomplete outcome documentation further hinder sustained progress [1-3,10,11,13].

To fully realize LaQshya’s potential, IGGMCH must prioritize capacity building, regular audits, and comprehensive quality monitoring to bridge implementation gaps and ensure long-term improvements in maternal and neonatal care [9,14-16]. Thus, the above findings definitely warrant frequent assessments and audits to improve the delivery of maternal health care services.

Strengths

The strength of this study lies in its comprehensive evaluation of the LaQshya program implementation at a high-volume tertiary care facility, IGGMCH. The study provides an in-depth analysis of key factors such as infrastructure upgrades, clinical protocol adherence, and patient satisfaction, offering valuable insights into the program’s effectiveness in improving maternal and neonatal care. By focusing on a large and diverse patient population with over 5,000 deliveries annually, the study effectively highlights the real-world challenges and successes of implementing such a large-scale quality improvement initiative. Furthermore, the use of both qualitative and quantitative data collection methods, including interviews, on-site observations, and patient feedback, adds depth to the findings, allowing for a more holistic understanding of the program’s impact. This thorough approach ensures that the study's conclusions are well-supported by evidence and applicable to similar settings across India.

Limitations

One of the primary limitations of the study is its reliance on a single healthcare facility, which may limit the generalizability of the findings to other settings, especially rural, low-load, or private healthcare centers. The study does not provide in-depth solutions or interventions to address these broader systemic issues, leaving critical gaps in the potential for immediate application of its findings. Additionally, while the study mentions infrastructural improvements and resource availability, it lacks detailed quantitative data on the extent of these improvements and how they directly correlate with maternal and neonatal outcomes. Another limitation is the potential bias in patient feedback due to the presence of hospital staff during interviews, which may have influenced responses. The potential Hawthorne effect from a single-day baseline observation may also have influenced staff behavior. Furthermore, the absence of a formal sample size calculation limits the ability to fully assess the adequacy of the participant pool, and the lack of outcome-linked health indicators restricts our capacity to evaluate long-term impact. Finally, although a before-and-after approach was adopted, the limited follow-up period constrains the ability to determine the sustainability of improvements over time. These expanded considerations better frame the interpretation of our findings and provide important context for applying them in other settings.

Recommendations

Based on the evaluation of LaQshya implementation at a tertiary healthcare facility in Nagpur, several key recommendations have emerged to strengthen and sustain the program’s impact.

Tackling Human Resource Shortages

One of the most pressing concerns is the shortage of trained professionals-particularly obstetricians, anesthetists, and nursing staff. To meet LaQshya’s goal of providing continuous, high-quality maternity care, the hospital must focus on recruiting and retaining skilled personnel. This can be supported through improved working conditions, appropriate incentives, and opportunities for professional growth.

Strengthening Clinical Protocol Adherence

While many staff members have undergone LaQshya training, consistent application of essential protocols-like partograph use, infection control, and active management of the third stage of labor-remains uneven. Refresher training sessions, supportive supervision, and routine audits can help reinforce protocol compliance and improve overall care quality.

Maintaining and Upgrading Infrastructure

Upgrades to labor rooms and maternity OTs have had a positive impact, but these changes need to be sustained. Regular inspection and calibration of medical equipment, along with continued focus on cleanliness, space, and privacy, are vital for creating a safe and respectful environment for both patients and providers.

Enhancing Quality Management Systems

Developing robust internal and external quality checks is essential. Periodic audits, detailed outcome tracking, and real-time assessments can help identify gaps early and guide necessary improvements based on data and clinical insight.

Building Staff Capacity

Ongoing training and competency assessments are critical. Structured skill-building programs, including simulations and hands-on workshops, can help healthcare teams stay updated and better equipped to manage complex situations effectively.

Listening to Patients

Patient satisfaction is central to LaQshya’s philosophy of respectful maternity care. Strengthening feedback mechanisms-such as anonymous forms or suggestion boxes-can help identify concerns quickly and foster a more responsive care environment.

Emphasizing Continuous Evaluation

Lastly, LaQshya’s effectiveness depends on regular and reflective evaluation. Institutionalizing structured assessments will ensure the program evolves with changing needs and that improvements are sustained over time.

These recommendations, if implemented, can help translate LaQshya’s vision into lasting improvements in maternal and newborn health-especially in high-volume, resource-constrained settings.

## Conclusions

The LaQshya program has enhanced maternal and neonatal care at IGGMCH, Nagpur, through infrastructure upgrades, improved clinical protocols, and better equipment availability, leading to safer care and improved patient experiences. However, persistent challenges-including shortages of skilled staff, inconsistent protocol adherence, and infrastructural gaps-limit full compliance with LaQshya standards. While privacy measures and communication have improved patient satisfaction, overcrowding and limited staff interaction remain concerns. Sustaining progress will require continued capacity building, rigorous quality monitoring, regular equipment inspections, and responsive patient feedback systems to address ongoing gaps and ensure long-term improvement.

## References

[1] LAQSHYA: Labour Room Quality Improvement Initiative 2017, National Health Mission. https://nhm.gov.in/index1.php.

[2] (2017). Ministry of Health and Family Welfare, Government of India. LaQshya-Labour Room Quality Improvement Initiative [Internet]. New Delhi: Government of India. https://nhm.gov.in/New_Updates_2018/NHM_Components/RMNCH_MH_Guidelines/LaQshya-Guidelines.pdf.

[3] Ronsmans C, Graham WJ (2006). Maternal mortality: who, when, where, and why. Lancet.

[4] Mahalakshmi M, Kanmani K, Kirubanidhi V, Swetha S (2023). LaQshya-an uphill climb: a review of implementation of LaQshya programme at a tertiary centre in Chennai. Int J Reprod Contracept Obstet Gynecol.

[5] Dorairajan G, Gopalakrishnan V, Chinnakali P, Balaguru S (2021). Experiences and felt needs of women during childbirth in a tertiary care center: a hospital-based cross-sectional descriptive study. J Obstet Gynaecol India.

[6] Devagappanavar G (2022). Assessment of labor room facilities in community health centers, Taluk hospitals, and the Gadag district hospital. Innov J Med Sci.

[7] WHO WHO (2019). WHO. Quality of care for maternal and newborn health: a monitoring framework for network countries [Internet]. Geneva: World Health Organization. Quality of Care for Maternal and Newborn-A Monitoring Framework for Network Countries.

[8] Sumankuuro J, Crockett J, Wang S (2018). Perceived barriers to maternal and newborn health services delivery: a qualitative study of health workers and community members in low and middle-income settings. BMJ Open.

[9] Singh SK, Kaur R, Gupta M, Kumar R (2012). Impact of national rural health mission on perinatal mortality in rural India. Indian Pediatr.

[10] WHO Recommendations: Intrapartum Care for a Positive Childbirth Experience (2018). World Health Organization. WHO Recommendations: Intrapartum care for a positive childbirth experience. Geneva: World Health Organization. WHO Recommendations: Intrapartum Care for a Positive Childbirth Experience.

[11] Singh S, Hasan Z, Sharma D, Kaur A, Khurana D, Shrivastava JN, Gupta S (2024). Appraising LaQshya's potential in measuring quality of care for mothers and newborns: a comprehensive review of India's Labor Room Quality Improvement Initiative. BMC Pregnancy Childbirth.

[12] Khan T, Mushtaq E, Khan F, Ahmad A, Sharma KA (2023). Decreasing the rate of surgical site infection in patients operated by cesarean section in a tertiary care hospital in India: a quality improvement initiative. Cureus.

[13] Karvande S, Sonawane D, Chavan S, Mistry N (2016). What does quality of care mean for maternal health providers from two vulnerable states of India? Case study of Bihar and Jharkhand. J Health Popul Nutr.

[14] Vincent V, Saha MK (2019). A study on evaluation of laqshay in Andaman. Rec Adv Path Lab Med.

[15] Dogne CG, Dudi J, Dogne N (2023). Perception of beneficiaries regarding quality of care and respectful maternity care being provided in delivery room using LaQshya guidelines. Indian J Med Spec.

[16] Pundappanavar BI, Kampli MS (2020). An Assessment of Status of Implementation and Functionality of LaQshya Initiatives in Karnataka, Maharashtra and Jharkhand. https://prc.mohfw.gov.in/fileDownload?fileName=197_LaQshya.pdf.

